# Aggressive Regimens for Multidrug-Resistant Tuberculosis Decrease All-Cause Mortality

**DOI:** 10.1371/journal.pone.0058664

**Published:** 2013-03-13

**Authors:** Carole D. Mitnick, Molly F. Franke, Michael L. Rich, Felix A. Alcantara Viru, Sasha C. Appleton, Sidney S. Atwood, Jaime N. Bayona, Cesar A. Bonilla, Katiuska Chalco, Hamish S. F. Fraser, Jennifer J. Furin, Dalia Guerra, Rocio M. Hurtado, Keith Joseph, Karim Llaro, Lorena Mestanza, Joia S. Mukherjee, Maribel Muñoz, Eda Palacios, Epifanio Sanchez, Kwonjune J. Seung, Sonya S. Shin, Alexander Sloutsky, Arielle W. Tolman, Mercedes C. Becerra

**Affiliations:** 1 Department of Global Health and Social Medicine, Harvard Medical School, Boston, Massachusetts, United States of America; 2 Partners In Health, Boston, Massachusetts, United States of America; 3 Division of Global Health Equity, Brigham and Women’s Hospital, Boston, Massachusetts, United States of America; 4 Socios En Salud-Sucursal Peru, Lima, Peru; 5 National Tuberculosis Strategy, Ministry of Health, Lima, Peru; 6 Tuberculosis Research Unit, Case Western Reserve University, Cleveland, Ohio, United States of America; 7 Massachusetts General Hospital, Boston, Massachusetts, United States of America; 8 Global Health Committee, Boston, Massachusetts, United States of America; 9 Hospital Nacional Sergio E. Bernales, Lima, Peru; 10 Center for Health Policy and Research, Massachusetts Supranational TB Reference Laboratory, Boston, Massachusetts, United States of America; University of Cape Town, South Africa

## Abstract

**Rationale:**

A better understanding of the composition of optimal treatment regimens for multidrug-resistant tuberculosis (MDR-TB) is essential for expanding universal access to effective treatment and for developing new therapies for MDR-TB. Analysis of observational data may inform the definition of an optimized regimen.

**Objectives:**

This study assessed the impact of an aggressive regimen–one containing at least five likely effective drugs, including a fluoroquinolone and injectable–on treatment outcomes in a large MDR-TB patient cohort.

**Methods:**

This was a retrospective cohort study of patients treated in a national outpatient program in Peru between 1999 and 2002. We examined the association between receiving an aggressive regimen and the rate of death.

**Measurements and Main Results:**

In total, 669 patients were treated with individualized regimens for laboratory-confirmed MDR-TB. Isolates were resistant to a mean of 5.4 (SD 1.7) drugs. Cure or completion was achieved in 66.1% (442) of patients; death occurred in 20.8% (139). Patients who received an aggressive regimen were less likely to die (crude hazard ratio [HR]: 0.62; 95% CI: 0.44,0.89), compared to those who did not receive such a regimen. This association held in analyses adjusted for comorbidities and indicators of severity (adjusted HR: 0.63; 95% CI: 0.43,0.93).

**Conclusions:**

The aggressive regimen is a robust predictor of MDR-TB treatment outcome. TB policy makers and program directors should consider this standard as they design and implement regimens for patients with drug-resistant disease. Furthermore, the aggressive regimen should be considered the standard background regimen when designing randomized trials of treatment for drug-resistant TB.

## Introduction

Multidrug-resistant strains of *Mycobacterium tuberculosis* accounted for approximately 5% of the 6.2 million tuberculosis (TB) cases notified in 2011. [1] Treatment for multidrug-resistant tuberculosis (MDR-TB) typically lasts between 18 and 24 months, and adverse events are common. [2] The combined frequency of cure and completion often remains below 65%. [3], [4], [5] Even when therapy is designed with access to the full complement of anti-TB agents presently available, outcomes rarely approach the target for TB treatment success (cure at least 85% of patients initiating therapy). [6], [7] The long duration and toxicity of current MDR-TB regimens are major obstacles to achievement of universal access to quality treatment. [8], [9] In addition, the poor outcomes seen with current regimens mean that, despite treatment, many MDR-TB patients will still develop chronic, highly resistant forms of TB that have a high mortality rate and can be transmitted to others. [10], [11].

For drug-resistant TB, improved treatment depends on introduction of new drugs and optimal use of existing drugs. Guidance about the use of drugs in MDR-TB regimens has been based on expert opinion, and most recently on GRADE methodology applied to available observational studies. [12], [13] Nevertheless, controversies persist about optimal regimen construction and duration. [6], [14], [15] Additional experience from observational treatment cohorts can inform the composition of optimal regimens.

One influential approach to the composition of regimens recommends a minimum of five drugs to which the isolate was documented or likely to be susceptible. This approach, to composing what we call an “aggressive” regimen, was presented in a 2004 article [16] and used as the foundation for WHO guidelines. [13], [17], [18], [19] To reach the five-drug minimum, the algorithm recommends inclusion of first-line agents, an injectable agent, a fluoroquinolone, and then any of the agents with documented bacteriostatic activity against *M. tuberculosis* including ethionamide/prothionamide, cycloserine/terizidone, and PAS. If a total of five likely effective drugs cannot be reached using these agents, the aggressive regimen also includes other agents of possible utility such as clofazimine, amoxicillin-clavulanate, and/or a macrolide antibiotic. This regimen was recommended to be delivered for 18–24 months past culture conversion, with the injectable agent being administered for 6 months after culture conversion.

Despite having had such an important impact on policy and guidelines, the effectiveness of this aggressive regimen–compared to the effectiveness of regimens not constructed according to this algorithm–has never been explicitly evaluated. The present retrospective, observational study evaluates whether this algorithm-based aggressive regimen was associated with a decreased rate of death when administered to patients with MDR-TB in Peru from 1999–2002. [16].

## Methods

### Study Population

The retrospective cohort included all patients who were enrolled between 1 February 1999 and 31 July 2002 in Lima, Peru, in ambulatory treatment for MDR-TB, which was tailored or individualized to each patient’s drug-susceptibility test (DST) results (N = 673). Patients were excluded from analysis either if (a) if the regimen delivered during the observational study period was not their first individualized MDR-TB treatment or (b) data on regimen composition were not available. We have previously reported on this cohort. [20], [21], [22], [23] Patients received care from a consortium led by the National TB Program. The consortium, which included the non-governmental organization Partners In Health (Socios En Salud-Sucursal Peru), scaled up this ambulatory program throughout metropolitan Lima during the study period.

### Treatment and Monitoring

Baseline evaluation, DST, and treatment monitoring were performed as described previously. [24] DST to the first-line drugs (isoniazid, rifampin, pyrazinamide, ethambutol, and streptomycin) was routinely performed. In more than 75% of patients, DST was also performed to the following second-line drugs: amikacin, capreomycin, cycloserine, ethionamide, kanamycin, *para*-aminosalacylic acid; ciprofloxacin or ofloxacin; and either gatifloxacin, levofloxacin, or moxifloxacin. Fewer than 25% of patients had isolates tested to other agents: amoxicillin-clavulanic acid, clofazimine, clarithromycin, or rifabutin. All patients who met the inclusion criteria specified above were included, regardless of the extent of DST performed on their infecting isolates.

Regimens were constructed using DST results and prior treatment exposure according to previously described principles. [16].

Outpatient treatment was directly observed, either at public-health centers or in patients’ homes, by community-health workers or nurses. Adverse events were managed by these workers according to established algorithms [25], [26] in consultation with physicians from the National TB Program consortium. Adjunct medical services (including thoracic surgery) and psycho-social and nutritional support were provided to patients free of charge as deemed necessary by expert providers. [24], [25], [27], [28], [29].

### Data Collection, Primary Exposure, Covariates and Outcome Definitions

Data were collected and recorded in a web-based electronic medical record during treatment. [30] A standardized paper chart abstraction was conducted to complete the dataset.

The primary exposure variable was receipt of an aggressive regimen. We classified the regimen as aggressive in the intensive phase if it contained at least five antituberculosis agents–including one of the injectable agents (streptomycin, kanamycin, capreomycin, amikacin) for at least six months after culture conversion and one fluoroquinolone–that met criteria suggesting efficacy according to the individual’s baseline DST and treatment history. The continuation phase of the aggressive regimen contained at least four likely effective oral drugs (including a fluoroquinolone). [16] An agent was considered efficacious if either (1) all *in vitro* sensitivity testing prior to the start of this regimen confirmed susceptibility to the agent used; or, (2) *in vitro* sensitivity testing to the agent was not available and the patient had not received the agent for >1 month prior to individualized treatment.

Exposure to an aggressive regimen was assessed for each treatment day because regimen adjustments could change exposure status. Changes occurred occasionally by design–regimens were started empirically and then adjusted when baseline DST results became available–and in response to adverse events, non-response to therapy, and drug stockouts. If at least 75% of regimen days in a month met the aggressive regimen definition, then the treatment month was coded as exposed; otherwise, the treatment month was coded as unexposed.

#### Covariates

Previous treatment was an important covariate abstracted from the record. There was significant variability in prior TB treatment regimens among the study participants. This included differences in numbers of prior regimens and contents of prior regimens: first-line drugs only or first- and second-line drugs; the latter were contained in the standardized regimen for MDR-TB (which was implemented by the National TB Program in 1997 [31]). To simplify, in the present analysis, prior TB treatment was dichotomized into two levels: (i) less prior treatment: 2 or fewer prior regimens, not including the standardized regimen for MDR-TB and (ii) more prior treatment: more than 2 regimens or prior treatment with the standardized regimen for MDR-TB.

Other variables collected include: demographics (age, sex, location of residence, treatment time period); all DST results at or prior to initiation of the individualized regimen (distribution of resistance is reported out of the 12 agents/drug classes listed above). Indicators of disease severity were collected including: hematocrit, nutritional status (body mass index [BMI] and clinical diagnosis of malnutrition), presence of extrapulmonary TB, respiratory difficulty (dyspnea or resting respiratory rate >26/minute), tachycardia (heart rate >100/minute), cavitary and bilateral disease on chest radiography. Comorbidities and risk factors–substance and tobacco use, human immunodeficiency virus infection, diabetes mellitus, cardiovascular and renal disease, and psychiatric and seizure disorders–were recorded.

#### Outcomes

Treatment outcomes (cure, completion, failure, death) were defined as previously described. [32] The primary study endpoint was time from initiation of the individualized regimen to death from any cause, while on treatment. Data were censored when an outcome other than death was recorded.

### Analysis

We modeled the association between receiving an aggressive regimen for at least 75% of the days in the current month and the hazard of death using Cox proportional hazards analysis. [33] Subjects were excluded from analyses if data were missing about the composition of the regimen.

Each covariate (prior treatment, sex, age, extent of disease on chest radiography, extent of resistance [number of drugs, XDR-TB], disease severity, and comorbidities) was evaluated for association with hazard of death.

Those variables that predicted the outcome at a p value ≤0.10 were considered candidates for the multivariate model. We retained a candidate variable in the final model if it remained associated with hazard of death at a p value ≤0.05 or if inclusion of that variable changed the effect estimate of aggressive in the model by ≥10%. We included age and sex in the final multivariable model due to their strong established link with poor outcomes from tuberculosis. We evaluated the possibility that the effect of an aggressive regimen was different in patients with confirmed extensively drug resistant TB (XDR-TB, TB caused by strains of *M. tuberculosis* resistant to at least isoniazid, rifampin, a fluoroquinolone, and a second-line injectable agent), compared to those without XDR-TB (effect modification), by including an interaction term in the multivariable analysis.

The proportional hazards assumption was tested by examining the interaction between the time-varying aggressive regimen variable and the treatment semester. Informative censoring was assessed by evaluating the association between default and the aggressive regimen. Missing values were multiply imputed using Markov Chain Monte Carlo methods to complete the dataset. All statistical tests were two-sided. Analyses were conducted using SAS version 9.12 (The SAS Institute, Cary, North Carolina).

This retrospective study was approved by the Committee on Human Studies at Harvard Medical School and by the Ministry of Health of Peru. Since the analysis was carried out using data that had been collected routinely under the aegis of the Peruvian National TB Program, informed consent was not required by the ethics boards.

## Results

Two patients were excluded because the regimen received during the study period was not their first individualized regimen for MDR-TB; two were excluded from analysis because details on the composition of their regimen were unavailable. Analyses were performed on data from 669 patients.

At initiation of the individualized regimen, patient isolates were resistant to a mean of 5.4 (SD 1.7) drugs; 48 (7.2%) had XDR-TB. One hundred seventy-three (25.9%) patients had received two or fewer previous regimens, not including the standardized regimen for MDR-TB; two patients had never received TB treatment. Respiratory difficulty (72.2%) and bilateral, cavitary disease (55.3%) were the most common indicators of severity. Documented HIV coinfection was rare, occurring in only 10 (1.5%) patients. Other comorbidities were more common: 233 (36.4%) patients had at least one other comorbidity (Table 1).

**Table 1:** Distribution of covariates at initiation of ITR.

COVARIATE	N = 669	Patients with specified characteristics
		N (%) or Mean (SD)
**PRIOR TREATMENT**		
Received ≤2 previous regimens without CER	668	173 (25.9)
**DEMOGRAPHICS**		
Female	669	261 (39.0)
Age^1^	668	31.4 (12.1)
Enrolled in Northern Lima	669	274 (41.0)
Enrolled prior to March 1, 2001	669	155 (23.2)
**INDICATORS OF SEVERITY**		
Bilateral, cavitary findings	637	352 (55.3)
Low BMI^2^ or malnutrition	573	225 (39.3)
Low hematocrit^3^	584	287 (49.1)
Tachycardia	652	196 (30.1)
Respiratory difficulty^4^	632	456 (72.2)
Extrapulmonary TB	668	60 (9.0)
Number of resistant agents^5^	669	5.4 (1.7)
Lab-confirmed XDR-TB^6^	669	48 (7.2)
Prior resective surgery	648	18 (2.8)
**COMORBITIES**		
Patients with at least one comorbidity^7^	640	233 (36.4)
HIV infection	656	10 (1.5)

1 Continuous variable, mean (standard deviation) presented.

2 <18.5 in women; <20 in men; or malnutrition established clinically.

3 ≤30% in women; ≤36% in men; when missing, also used hemoglobin ≤10 in women and ≤12 in men.

4 Dyspnea; resting respiratory rate greater than 26/minute.

5 Resistance to the following 12 drugs or drug classes was tested: capreomycin, cycloserine, ethambutol, ethionamide, isoniazid, kanamycin or amikacin, PAS, pyrazinamide, rifampicin, streptomycin, first-generation fluoroquinolones (ciprofloxacin, ofloxacin), and later-generation fluoroquinolones (gatifloxacin, levofloxacin, moxifloxacin).

6 Isolate resistant to at least isoniazid, rifampin, fluoroquinolone, and injectable (kanamycin, capreomycin, or amikacin).

7 This includes the following comorbidities: cardiovascular disease (12), diabetes mellitus (18), hepatitis or cirrhosis (10), epilepsy/seizures (11), renal insufficiency (7), psychiatric disorder (116), ever smoked (66), ever used/abused alcohol or other substance (52).

The median duration of the regimen was 24.4 (inter-quartile range [IQR]: 19.4–27.8) months. Among those who received an aggressive regimen for at least one month (547 [82%]), the median duration of that regimen was 21 (IQR: 15–26) months. Of note, among the 48 patients with confirmed XDR-TB, 28 (58%) received an effective regimen during at least one month.

Outcomes were available for 665 patients. Cure or completion was achieved in 442 (66.1%) while death occurred in 139 (20.8%) (Table 2).

**Table 2:** Treatment outcomes of 669 patients enrolled in individualized treatment for MDR-TB in Peru between February 1999 and July 2002. (Adapted from Mitnick et al., 2008) [20].

Outcome	N (%)
**Cured/Completed**	442 (66.1)
**Treatment Failed**	17 (2.5)
**Died**	139 (20.8)
**Defaulted**	67 (10.0)
**Missing/Transferred Out**	4 (0.6)
**Total**	669 (100)

In a time-varying univariate analysis, receiving more than 75% of doses of an aggressive regimen in a month was associated with a decreased hazard of death (HR: 0.62; 95% CI: 0.44,0.89). Less prior treatment at baseline was also associated with decreased rate of death (p<0.01). Baseline characteristics–increased age, bilateral and cavitary disease on chest radiography, a number of indicators of severity, comorbities (other than HIV), and HIV–were all significantly associated with increased rate of death (p<0.05). XDR-TB was not associated with any elevated risk (p = 1.00); however an increase in the number of drugs to which the isolate was resistant was associated with increased rate of death (p<0.01) (Table 3). In multivariable analysis (Table 4), exposure to an aggressive regimen in a month was independently associated with decreased rate of death (HR: 0.63; 95% CI: 0.43,0.93); in all semesters of treatment, the effect of an aggressive regimen remained protective. Less prior TB therapy (HR: 0.43; 95% CI: 0.25,0.74) was also associated with decreased rate of death. Low BMI (HR: 2.45; 9 5% CI: 1.63,3.68), and tachycardia (HR: 2.19; 95% CI: 1.50,3.19) were independently associated with increased rate of death. Women were also at increased risk of death (HR: 1.45; 95% CI: 1.02,2.07). When we compared the benefit of receiving an aggressive regimen in patients with confirmed XDR-TB and those without XDR-TB, we found no difference. Therefore, the interaction term was excluded from the final multivariable model. Censoring due to default was not associated with exposure to the aggressive regimen.

**Table 3:** Univariate, time-varying Cox proportional hazards analysis of aggressive regimen and time to death.

COVARIATE	Hazard ratio, univariate analysis	95% CI, univariate analysis	p-value
Monthly exposure to aggressive regimen	0.62	0.44, 0.89	0.01
**PRIOR TREATMENT**			
Received ≤2 previous regimens without CER	0.36	0.21, 0.61	<0.01
**DEMOGRAPHICS**			
Female	1.25	0.89, 1.76	0.19
Age^1^	1.02	1.00, 1.03	0.01
Enrolled in Northern Lima	0.71	0.50, 1.01	0.06
Enrolled prior to March 1, 2001	1.11	0.74, 1.66	0.63
**INDICATORS OF SEVERITY**			
Bilateral, cavitary findings	2.15	1.46, 3.16	<0.01
Low BMI^2^ or malnutrition	4.29	2.89, 6.36	<0.01
Low hematocrit^3^	2.24	1.53, 3.27	<0.01
Tachycardia	3.21	2.29, 4.49	<0.01
Respiratory difficulty^4^	4.70	2.54, 8.72	<0.01
Extrapulmonary TB	2.82	1.84, 4.33	<0.01
Number of resistant agents^5^	1.17	1.06, 1.28	<0.01
Lab-confirmed XDR-TB^6^	1.00	0.54, 1.86	1.00
Prior resective surgery	1.49	0.61, 3.65	0.38
**COMORBITIES**			
Patients with at least one comorbidity^7^	1.99	1.41, 2.81	<0.01
HIV infection	3.16	1.29, 7.74	0.01

1 Continuous variable, mean (standard deviation) presented.

2 <18.5 in women; <20 in men; or malnutrition established clinically.

3 ≤30% in women; ≤36% in men; when missing, also used hemoglobin ≤10 in women and ≤12 in men.

4 Dyspnea; resting respiratory rate greater than 26/minute.

5 Resistance to the following 12 drugs or drug classes was tested: capreomycin, cycloserine, ethambutol, ethionamide, isoniazid, kanamycin or amikacin, PAS, pyrazinamide, rifampicin, streptomycin, first-generation fluoroquinolones (ciprofloxacin, ofloxacin), and later-generation fluoroquinolones (gatifloxacin, levofloxacin, moxifloxacin).

6 Isolate resistant to at least isoniazid, rifampin, fluoroquinolone, and injectable (kanamycin, capreomycin, or amikacin).

7 This includes the following comorbidities: cardiovascular disease (12), diabetes mellitus (18), hepatitis or cirrhosis (10), epilepsy/seizures (11), renal insufficiency (7), psychiatric disorder (116), ever smoked (66), ever used/abused alcohol or other substance (52).

**Table 4:** Multivariable, time-varying Cox proportional hazards analysis of aggressive regimen and time to death.

Variable	Hazard ratio, multivariable analysis	95% CI, multivariable analysis
**Monthly exposure to aggressive regimen**	0.63	0.43, 0.93
**Received ≤2 previous regimens without CER**	0.43	0.25, 0.74
**Female**	1.45	1.02, 2.07
**Age**	1.01	1.00, 1.03
**Low BMI or malnutrition**	2.45	1.63, 3.68
**Tachycardia**	2.19	1.50, 3.19
**Extrapulmonary TB**	1.68	1.05, 2.68
**At least one comorbidity, other than HIV**	1.71	1.21, 2.43
**HIV Infection**	2.72	1.03, 7.24
**Number of resistant agents**	1.03	0.92, 1.15

## Discussion

Here we test the utility of an operational definition of an aggressive regimen for treating MDR-TB. We previously proposed an algorithm for MDR-TB regimen design; this entailed, preferentially, any first-line drugs to which the isolate was sensitive, an injectable for at least 6 months after culture conversion, a fluoroquinolone, and a complement of bacteriostatic second-line drugs to reach the target of five. [16] In accordance with this algorithm, for the present analysis, we specified that an aggressive regimen had the following characteristics: at least five likely efficacious drugs, including a fluoroquinolone and injectable in the intensive phase; in the continuation phase, the requirement was at least four likely efficacious drugs, including a fluoroquinolone. Exposure to an aggressive regimen was time-varying due to drug changes during the course of treatment. Ignoring this variability, or requiring a minimum duration of exposure to the regimen for classification as aggressive, would result in misclassification of exposure. [34] The consequence could be biased effect estimates, in some cases overestimating the benefits of an aggressive regimen. [35] We therefore evaluated the effect of monthly exposure to an aggressive regimen on death rate.

In this large, well-characterized treatment cohort in Peru, we found that receipt of a regimen that met all of these criteria was a robust predictor of successful MDR-TB treatment outcome in the face of all measured covariates. These results complement our recent finding that receipt of an aggressive regimen for at least 18 months was associated with a lower rate of recurrent TB. [23] Even after controlling for risk factors such as extensive prior anti-TB treatment, advanced age, poor nutritional status, and indicators of advanced disease such as tachycardia, the rate of death was nearly halved in each month in which patients received an aggressive regimen. When we compared the small group of patients with XDR-TB to the others, we found that, unsurprisingly, aggressive regimens were less likely to be constructed in the XDR-TB group. However, most (31) XDR-TB patients could receive at least one injectable to which their isolate was not confirmed to be resistant and all could receive at least one fluoroquinolone to which their isolate was not resistant. The benefits associated with receiving an aggressive regimen were also observed in the XDR-TB group.

It is noteworthy that several other factors were independently associated with death. These included having received extensive prior treatment–at least 2 previous treatments with or without the standardized regimen for MDR-TB. This effect may have been mediated through resistance, which is known to be a consequence of repeated TB treatment. [36] Receipt of fewer prior regimens reduced the rate of death by almost half and likely reflects, in part, less resistance. And, avoiding ineffective regimens can result in less disease severity–also independently associated with increased rate of death–and less cumulative toxicity from anti-TB treatment. For all these reasons, and to preserve program resources, minimizing exposure to inadequate regimens should be a priority of TB treatment programs. To this end, current program policy in Peru is to screen all TB patients for resistance; [37] this is a change from the policy in place at the time the present study was conducted, which called for resistance testing only after failure of at least two TB regimens. Additional attention to gender–among other social determinants of health–and MDR-TB may also be necessary since these findings corroborate our earlier non-significant findings of increased risk of poor outcomes among women with MDR-TB [24] but stand in contrast to those of a recent meta-analysis, which found an increased risk of poor outcomes among men. [38] Lastly, attention to improved diagnostics and treatment of HIV coinfected patients and patients with extrapulmonary MDR-TB is indicated by this study. None of these patients was receiving ART. These findings build on those that have previously identified an increased risk of mortality among MDR-TB patients with HIV coinfection. [39], [40] Prior work has demonstrated that this increased risk can be reduced by starting ART in dually infected populations; [41], [42] the present study adds that benefits may also accrue through use of aggressive regimens for MDR-TB.

In addition, the algorithmic approach examined in this study provides useful guidance for TB programs and supplements global guidelines. Current WHO recommendations [13], [43] call for “…four second-line anti-tuberculosis drugs likely to be effective (including a parenteral agent), as well as pyrazinamide…” This was based on an analysis that tried to elucidate the role of individual drugs and a simple minimum number of drugs. Approaching the problem differently, our analysis revealed that a regimen containing five likely effective drugs reduced the risk of death in this population of patients with extensive prior treatment. Use of this algorithmic approach may provide additional options for composing aggressive regimens in settings in which the specific drugs recommended in the Guidelines are not available, or when pyrazinamide is not a likely effective drug.

Improving the success of MDR-TB treatment with existing drugs is essential. Results achieved to date have been far from optimal: pooled estimates indicate that only 62% (57%–67%) of patients treated had favorable outcomes. [38], [44].

With multiple new compounds in the drug-development pipeline, truly optimized regimens are also critical as comparator background regimens in trials. The consequence of use of a *sub-optimal* background regimen for MDR-TB treatment was illustrated in a study of a new anti-TB agent: only 9% of the placebo group experienced sputum culture conversion after two months of treatment; [45] this is considerably lower than in other reports of MDR-TB treatment. [46], [47] Although there may be other explanations for this relatively low conversion frequency (i.e., MGIT culture system, more extensive parenchymal damage), insufficient efficacy of the background regimen cannot be excluded.

The aggressive regimen described in the present study could be used as a background regimen and comparator in studies of new drugs. This would afford greater protection to patients with more extensive prior exposure than does a standardized regimen whose efficacy was demonstrated in a population without prior exposure to second-line drugs or known HIV infection. [48] The quality of studies of new drugs to treat MDR-TB will be enhanced immediately by incorporating novel evidence such as that we report here, which can guide construction of an optimized background regimen.

As in other retrospective studies, the potential for unmeasured confounding exists. Since patients were not deliberately (randomly or otherwise) assigned to non-aggressive regimens, we cannot rule out the possibility that the patients receiving and not receiving aggressive regimens differed in ways that also influenced the risk for death. Controlling for indicators of disease severity and comorbidities likely reduced the possibility of such confounding. Adverse events, however, were not recorded routinely and may have been linked both to the inability to construct an aggressive regimen and to the risk of death. This potential link should be evaluated in future, prospective studies. With respect to the exposure variable, although its time-varying assignment reduces misclassification, some potential for misclassification remains. This is because the definition of aggressive allows that drugs not previously received, and for which sensitivity testing had not been performed, are considered to contribute to an aggressive regimen. This risk of misclassification is greatest for the fluoroquinolones and injectables for which there is at least partial cross-resistance among members of the class. [49], [50], [51], [52], [53] If treatment months are misclassified as aggressive when they contain drugs to which patient isolates are resistant, this would likely bias the effect estimate towards the null (i.e., make the aggressive regimen seem less protective than it is). Lastly, we note that this study was conducted in a population with significant prior treatment exposure, and may represent a survival cohort. It is impossible, however, to assess the effect of survivor bias on treatment outcomes without having a comparison group–that is, MDR-TB patients who had not received prior treatment; of note only two patients in this study had received no prior treatment for TB. Since, under program conditions, therapy for MDR-TB is often reserved for patients who have received repeated treatments for TB, our results can be generalized to many patient populations treated in low- to middle-income countries. And, as noted above, our results indicate that prior exposure should be limited in order to facilitate composition of an aggressive regimen and reduce the risk of death.

In conclusion, these findings support the early use of an aggressive regimen for MDR-TB. The use of such regimens improves patient outcomes and is essential to stem the epidemic of multi-drug resistance, which affects roughly one-half million new TB patients annually. [54], [55], [56] Treating MDR-TB patients with sub-standard regimens likely fuels the development of even more resistant strains, leading to the predictable tragedy making news most recently: strains resistant to all drugs tested. [57], [58], [59].
